# An empirical study of exploring the predictors of university teachers’ job satisfaction in Bangladesh: A structural equation modeling approach

**DOI:** 10.1016/j.heliyon.2025.e41740

**Published:** 2025-01-06

**Authors:** Md Abdul Hye Zebon, Abdus Sattar, Md Sazzadur Ahamed

**Affiliations:** a Department of Software Engineering, Daffodil International University, Dhaka, Bangladesh; b Department of Computer Science and Engineering, Daffodil International University, Dhaka, Bangladesh

**Keywords:** Salary and financial benefits, Career growth and opportunity, Working environment, Relationship with colleagues, Leadership, Recognition, University teachers, Job satisfaction, PLS-SEM, Bangladesh

## Abstract

This study aims to investigate the factors influencing job satisfaction among university teachers, considering various complex constructs such as salary and financial benefits, career growth and opportunities, relationships with colleagues, recognition, working environment, and leadership. Utilizing a quantitative cross-sectional research design, the present study was conducted in Bangladesh between August and December 2022. Encompassing 7 public universities and 12 private universities, the research purposively sampled 95 participants, adhering to a systematic and comprehensive approach to data collection. Data were analyzed using Partial Least Squares Structural Equation Modeling (PLS-SEM). The empirical findings of the study revealed that career growth and opportunities (β = 0.225, t = 2.023, p < 0.10), leadership (β = 0.262, t = 1.757, p < 0.10), and relationships with colleagues (β = 0.166, t = 1.519, p < 0.10) had a significant positive impact on university teachers' job satisfaction. On the other hand, salary and financial benefits (β = 0.082, t = 0.823, p > 0.10), recognition (β = 0.02, t = 0.208, p > 0.10), and working environment (β = 0.14, t = 1.057, p > 0.10) had a positive but less significant impact on job satisfaction among university teachers. The overall findings provide valuable insights that can assist policymakers and stakeholders in implementing synchronized strategies to enhance job satisfaction among university teachers, ultimately leading to improved university teachers’ job performance.

## 1 Introduction

Education, being one of the basic human rights, is very important for the development of a society as well as a nation. Every person is entitled to this fundamental human right since quality education is a requirement for any human resource development. A group of highly qualified teachers is necessary for a successful educational system. Therefore, to build an educated nation, finding and training high-quality teachers is essential. Education is a dynamic process that helps people develop new habits, attitudes, beliefs, and abilities [1]. According to the International Standard Classification of Schooling (ISCED), there are nine stages of formal educational achievement, starting with early childhood education and going all the way up to a Ph.D. degree [2]. Ensuring proper and quality education is very important as a country's ability to reduce poverty and build its economy sustainably is directly impacted by education [[3], [4], [5], [6]]. However, because of its complicated structure and difficulty measuring complexity, defining education quality is confounding and sometimes difficult to assess. The National Center for Education Statistics (NCES) [7] recognized teachers as one of the important markers of educational institution quality to clarify why some educational institutions may be better than others. Teaching is widely considered to be one of the most important and well-respected professions due to its significant impact on students’ success and the overall quality of education [8]. Finding and keeping skilled teachers is a critical aspect of teacher recruitment worldwide. However, there is a global concern rising about the lack of qualified teachers and high teacher turnover rates. A study [9] shows that developed countries like the USA are facing a great turnover rate in the teaching profession with up to 50 % of new teachers quitting the teaching profession within 5 years. A significant number of teachers, more than 33 %, had high burnout with low job satisfaction, low self-efficiency, and low professional commitment, according to a recent study [10].

In addition, due to the fact that work performance is directly influenced by job satisfaction, it has become a crucial factor to focus on teachers’ job satisfaction as well. In a study by Amin et al. [11], the authors used five aspects to measure job satisfaction: work, salary, supervision, promotion opportunity, and relationship with fellow workers. Using these five aspects, the authors concluded that there is a positive correlation between teachers’ job satisfaction and job performance. It follows that ensuring teachers' satisfaction with their jobs is crucial if we want to improve education quality. The aspects that affect teachers' job satisfaction, such as the working environment, syndrome of burnout, self-reliance, inspiration, efficiency, retention, etc., have therefore been the topic of research. Researchers are attempting to learn more about the factors that affect teachers' job satisfaction since these teachers are less likely to quit and more likely to deliver excellent teaching.

When it comes to measuring job satisfaction in the teaching profession, several factors are impactful and should be considered. Over the years, there have been a lot of studies done around the world to measure job satisfaction in this profession. In a recent study by BURÓN et al. [12], 5 job facets were used to measure job satisfaction for teachers, in which Wage or Salary was listed as one of the top facets. Similarly, authors [13] used a 9-factor based subscale to measure teachers’ job satisfaction, where the salary was one of the top ranking factors. Whereas Ghenghesh [14] used 11 variables to determine the source of teachers’ job satisfaction and 12 variables to determine the source of teachers’ job dissatisfaction where the salary was one of the top variables for measuring both teachers’ job satisfaction and dissatisfaction. Also, similar studies [[15], [16], [17], [18], [19], [20], [21]] used salary or financial benefits to measure job satisfaction. The significance of salary and financial benefits as a determinant of job satisfaction among teachers in Bangladesh is underscored by its multifaceted implications. Beyond the immediate monetary remuneration, this factor encapsulates the broader financial considerations integral to a teacher's professional fulfillment. It encompasses not only the base salary but also additional allowances, benefits, and perceived financial stability. Therefore salary and financial benefits can be considered one of the most important factors in measuring job satisfaction in the teaching profession where a good pay scale can ensure higher job satisfaction and lower turnover rates, and a bad pay scale can lead to frustration and low job satisfaction among teachers.

Another factor to be considered is career growth and opportunities. Studies show [13,14] that opportunity for promotion can be an impactful factor while measuring a teacher’s job satisfaction, due to the fact that authors listed this as one of the top 10 factors for teachers’ job satisfaction. Sultana et al. [15] concluded that promotion is one of the responsible factors for the job satisfaction of school teachers in Bangladesh. On the other hand, Masum et al. [21] concluded career growth to be one of the important determinants of academic job satisfaction in private universities in Bangladesh. Other studies [[16], [17], [18], [19], [20]] used similar factors like career growth, personal growth, training opportunity, and opportunity for higher education to measure job satisfaction in the teaching profession. The prominence of career growth and opportunities as a pivotal factor influencing job satisfaction among university teachers in Bangladesh extends beyond the immediate professional landscape. This facet encompasses the prospect of professional advancement, continuous learning, and the availability of avenues for career development. It encapsulates not only vertical progression within the academic hierarchy but also lateral opportunities for skill enhancement and diversification. Offering opportunities for professional development and career advancement can have a positive impact on teacher job satisfaction. When teachers feel valued and invested in their work, they are more likely to be motivated to provide high-quality teaching and student support, leading to a better educational experience for students.

A study by Ali et al. [18] used multiple factors like the classroom, office room, personal room, washroom, and university location separately which represents the working environment as a whole to measure teachers’ job satisfaction. Similar to this, a model was developed to assess the job satisfaction of teachers at private universities in Bangladesh, where one of the key determinants was the working environment [21]. The authors came to the conclusion that stress-free workplace conditions might lead to higher levels of job satisfaction among employees. Studies [15,22] show that the working environment is a key factor in measuring teachers’ job satisfaction. This factor, which is essential to the academic environment, includes the physical location of the university, the layout of the campus, the design of the personal work areas, the organization of the office spaces, the quality of the classrooms, the upkeep standards of the restroom facilities, and, most importantly, the workload distribution and nature. A positive and supportive working environment is essential for teacher job satisfaction. This can lead to better educational outcomes for students and a more positive and productive working environment for teachers.

Relationships with colleagues can be very impactful while measuring the job satisfaction of a teacher. Mertler et al. [17] used 18 variables to determine teachers’ job satisfaction among which interpersonal relationship with colleagues was ranked in the top 10 factors. Similarly [23], authors concluded that a positive relationship between school administration and teachers leads to a reduction in teacher dissatisfaction. Also, other studies [[18], [19], [21]] used relationships with colleagues as a major factor for measuring the job satisfaction of a teacher. This factor involves the quality and nature of interactions among faculty members, fostering a collaborative and supportive atmosphere within the institution. Relationships with colleagues encompass the exchange of ideas, mutual support in professional endeavors, and a congenial work culture. Positive relationships with colleagues are essential for teachers’ job satisfaction. By fostering a culture of collaboration, mutual support, and respect, educational institutions can create an environment where teachers feel invested, engaged, and empowered in their work. This, in turn, can lead to better educational outcomes for students and a more positive and productive working environment for teachers.

The relationship between variables that affect teacher satisfaction, such as leadership, school environment, communication and engagement, structure, and work satisfaction, was studied by Crisci et al. using the Partial Least Square Path Modelling (PLS-PM) method [23]. The study discovered that encouraging teacher work happiness requires strong leadership. The authors also concluded that a positive relationship between institution administrations can reduce teacher dissatisfaction. In a separate study [24], Liu et al. focused on two commonly studied leadership models in education: distributed leadership and instructional leadership. The research found that both models have a positive impact on teacher job satisfaction by fostering a supportive institutional culture. Other studies [18,19] also used leadership as an impactful factor while measuring teachers’ job satisfaction. Within the parameters of this study, leadership pertains to the intricate dynamic between teachers and the managerial and administrative entities within the university. This construct encompasses the nuanced interplay of interpersonal relationships, communicative efficacy, and administrative support. The leadership of an educational institution plays a critical role in teacher job satisfaction. By providing clear and consistent communication, adequate support and resources, and a culture of respect and professionalism, administrators can help to create a positive and engaging work environment for teachers. This, in turn, can lead to better educational outcomes for students and a more productive and effective educational institution.

Finally, perhaps the most important factor in measuring the job satisfaction of a teacher is recognition. Study [[14], [16], [17], [18], [19]] shows that the social status that comes with being a teacher is an impactful factor while measuring the job satisfaction of a teacher. Also, the recognition for the work of a teacher from the administration is important for the teacher. Recognition encapsulates the extent to which educators are esteemed within both the academic and wider community. It involves the acknowledgment of their scholarly endeavors, teaching proficiency, and overall commitment to the educational landscape. By providing regular and meaningful recognition for the contributions and efforts of teachers, educational institutions can create a positive and engaging work environment that promotes motivation, empowerment, and job satisfaction. This, in turn, can lead to better educational outcomes for students and a more productive and effective educational institution.

To provide a comprehensive understanding of job satisfaction among university teachers, it is essential to consider the extensive body of literature addressing various factors influencing this phenomenon. Prior studies have examined job satisfaction through different lenses, including the Job Demands-Resources (JD-R) model, which highlights the impact of job demands and resources on university teachers' well-being and job satisfaction [25]. Additionally, the adoption of blended learning approaches in higher education has been shown to influence lecturers' perceptions and job satisfaction, emphasizing the importance of technology integration and institutional support [26,27]. Research on teacher attrition underscores the significant relationship between job satisfaction, burnout, and teachers' intentions to quit, revealing that while job satisfaction can mitigate attrition, burnout remains a predominant factor leading to teacher turnover [28]. Furthermore, the implementation of performance management systems, along with training and development initiatives, has been identified as crucial in enhancing job satisfaction among educators, particularly in low-income contexts [29]. These findings collectively underscore the multifaceted nature of job satisfaction among university teachers, necessitating a holistic approach to address various influencing factors and enhance overall job satisfaction and performance.

While teacher job satisfaction is critical in preserving excellent education, growing teacher turnover rates and a worsening standing in the teaching profession have had a detrimental influence on overall learning outcomes, according to the findings of the aforementioned research. As a result, there is a global lack of qualified teachers. According to the University Grants Commission of Bangladesh (UGC), there are 53 public universities and 109 private universities currently active in Bangladesh that offer various Bachelor’s Degrees [30,31]. Although developed countries are always seeking to measure teacher job satisfaction by frequent observation, there have been a few research [15,[18], [19], [20], [21],^32^] identified in the context of Bangladesh. In addition, most of the above studies used qualitative research methods to explore the characteristics that influence teacher job satisfaction, however, the significant constraints that may be investigated in the future are a lack of quantitative research, neglecting essential variables, a limited sample size, and a lack of comprehensiveness. To bridge this gap, the key purpose of the study is to determine the key affecting factors of university teachers’ job satisfaction. To this end, this study empirically examines the determinants of job satisfaction among university teachers in Bangladesh. It will situate itself at the nexus of educational language policy and human resources management, providing an analytical lens to scrutinize a critical intersection. The findings will be poised to yield substantial implications for legislative bodies, educational administrators, and university faculty, particularly within the context of developing nations. This academic inquiry posits that the factors influencing job satisfaction may exhibit distinctive characteristics in developing countries, thereby offering novel insights for the formulation of effective policies and administrative practices. Consequently, this study will answer the following questions.

RQ1 What are the predictors of university teachers’ job satisfaction in Bangladesh?

RQ2 How the predictors are affecting the overall job satisfaction of university teachers in Bangladesh?

RQ3 Which factors have a significant impact on the overall job satisfaction of university teachers in Bangladesh?

These research questions are aligned with the study’s primary objectives, which are summarized in Table 1.

**Table 1:** Research objectives and questions.

Objective	Research Question
1. Identify the predictors of university teachers’ job satisfaction in Bangladesh.	RQ1: What are the predictors of university teachers’ job satisfaction in Bangladesh?
2. Understand how these predictors influence overall job satisfaction.	RQ2: How do these predictors affect the overall job satisfaction of university teachers in Bangladesh?
3. Determine which factors have the most significant impact on job satisfaction.	RQ3: Which factors have a significant impact on the overall job satisfaction of university teachers in Bangladesh?

## 2 Materials and methods

### 2.1 Conceptual model and hypotheses

**Fig. 1:**
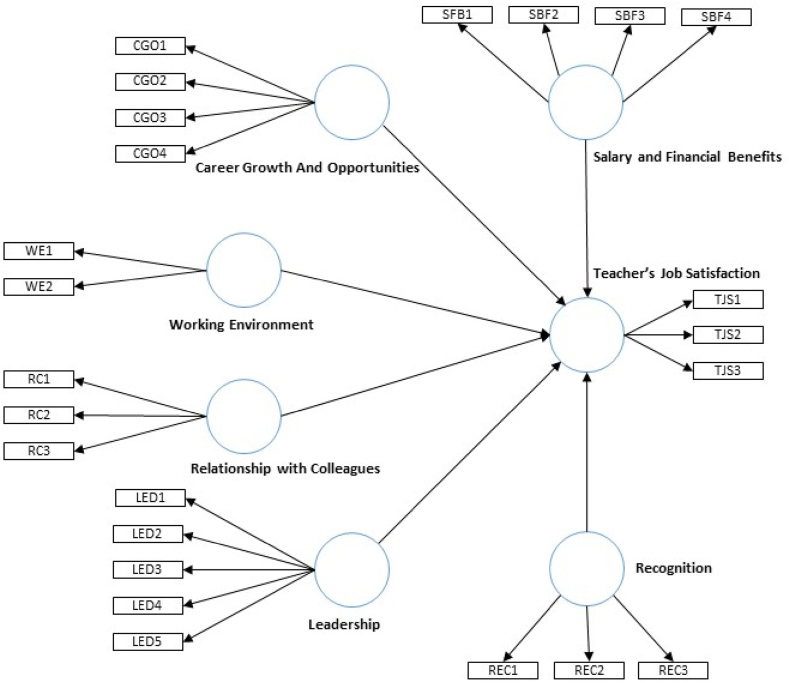
Conceptual framework.

In this study, a conceptual model is suggested and put to the test in order to analyze the predictive factors of university teachers’ job satisfaction (see Fig. 1). Seven constructs are included in it: (i) salary and financial benefits, (ii) career growth and opportunity, (iii) working environment, (iv) relationship with colleagues, (v) leadership, (vi) recognition, and (vii) teachers’ job satisfaction. Six of the constructs (i-vi) are exogenous (independent), while one (vii) is endogenous (dependent). Decisions regarding the inclusion of latent variables or constructs and directional causal relationships within the conceptual model were supported by existing theoretical considerations from the previous studies [[10], [11], [12], [13], [14], [15], [16], [17], [18], [19], [20], [21], [22], [23], [24], [32]]. Finally, the study hypotheses can be developed as follows in accordance with the conceptual model shown in Fig. 1.

H_1_: Salary and financial benefits positively affect teachers’ job satisfaction.

H_2_: Career growth and opportunities positively affect teachers’ job satisfaction.

H_3_: Working environment positively affects teachers’ job satisfaction.

H_4_: Workload negatively affects teachers' job satisfaction.

H_5_: Good relationships with peers positively affect teachers’ job satisfaction.

H_6_: Good relationships with leadership positively affect teachers’ job satisfaction.

H_7_: Recognition of work positively affects teachers' job satisfaction.

### 2.2 Study design and settings

The research methodology used in this study was a quantitative cross-sectional approach, and the study was carried out in 7 public universities and 12 private universities in Bangladesh from August to December 2022. The cross-sectional design was chosen due to its efficiency in capturing a snapshot of the current state of job satisfaction among university teachers within a specific time frame, which is useful for identifying and analyzing prevalent trends and correlations [33]. While longitudinal studies can provide insights into changes over time, the cross-sectional approach is more practical and cost-effective for this exploratory phase, allowing for a broad comparison across different types of institutions [34]. Additionally, given the time and resource constraints, a cross-sectional design was deemed appropriate to gather immediate data to inform policy and practice [35].

### 2.3 Population and sample size

**Fig. 2:**
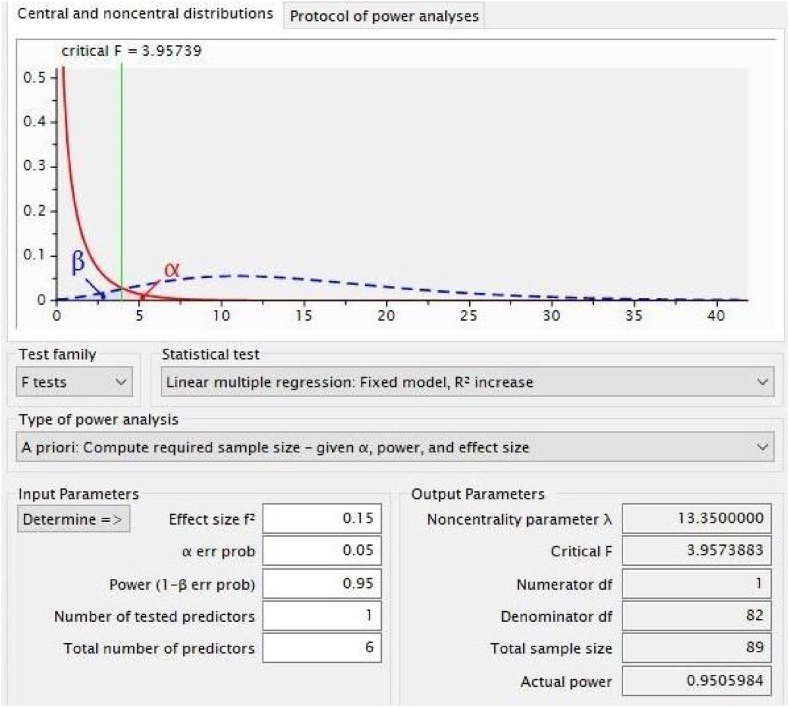
Power result of required sample size.

According to a yearly report released by the UGC in 2022 [36], the target population of this study is the 165,123 teachers employed by both private and public universities in Bangladesh. However, 95 randomly chosen teachers made up the study's sample size. Despite the sample size appearing to be small, this study used Partial Least Squares Structural Equation Modeling (PLS-SEM), an established multivariate analytical approach [37,38]. Despite having a small sample size, PLS-SEM is well renowned for having excellent levels of statistical power. The PLS-SEM sample size estimation method is based on a number of benchmarks. A common example of this is the "10 times rule," which specifies that the sample size must be at least ten times the indicators used to evaluate the construct [39]. The studies conducted in Refs. [40,41] revealed, however, that the 10 times rule can provide radically erroneous predictions of the minimal sample size required. The present study used G ∗ Power (version 3.1.9.4) [42], a regularly used [43,44] and recommended [38] tool, to find out the minimal sample size. Since the proposed conceptual model of this study has six components (predictors of teachers’ job satisfaction), the authors chose the effect size (f2) as medium 0.15, err prob as 0.05, and power (1- err prob) as 0.95 (see Fig. 2). These choices are in line with previous research [[38], [39], [42], [43]]. The computation ultimately produced the needed sample size of 89. Thus, the 95 samples in this investigation are sufficient to assure acceptable statistical power.

### 2.4 Pilot study

Prior to the questionnaire being made available to a broader audience, a pilot study was conducted with 10 university teachers, including 5 from public universities and 5 from private universities in Bangladesh. The participants' ages ranged from 30 to 55 years, with a mean age of 42.3 years (standard deviation = 7.6 years). This diverse group of participants was chosen to ensure a representative sample of the target population.

The pilot study aimed to evaluate the clarity, relevance, and comprehensiveness of the questionnaire. Participants were asked to complete the questionnaire and provide feedback on any items they found confusing or ambiguous. Based on their responses and suggestions, modifications were made to the questions on section 4, 5 [Appendix-A] to enhance clarity and ensure all questions were easily understood. For example, some technical terms were simplified, and instructions for certain sections were made more explicit.

To assess the reliability of the revised questionnaire, Cronbach's alpha (α) test was performed. The results indicated that the majority of the items had good internal consistency, with Cronbach's alpha values exceeding 0.75 (α > 0.75) for most sections. This high level of reliability confirms the internal consistency of the questionnaire items, as shown in equation (1)[45].

(1) $$\alpha =\frac{N\underline{c}}{\underline{v\;}+\left(N-1\right)\underline{c}}$$

Here, α = Cronbach's alpha, $\underline{c}$ = average inter-item covariance, N = number of items, $\underline{v\;}=$ average variance.

The pilot study provided valuable insights that informed the final adjustments to the questionnaire, ensuring it was well-suited for the main study's objectives and the target population.

### 2.5 Data collection instrument and procedure

Between August and December 2022, teachers from 19 universities provided the primary data for this study. Experts in quantitative research prepared the questionnaire and no existing instrument(s) were followed, which can be found in Appendix A, based on the factors that influence teachers' job satisfaction (e.g., salary and financial benefits, career growth and opportunity, working environment, relationship with colleagues, leadership, and recognition). It included 30 closed-ended questions in total, divided into two main groups. In the first portion, the teacher's basic information, including age, department, level of education, etc. was to be gathered. 24 items in the second part of the survey were devoted to examining the variables that affected teachers' job satisfaction. With options ranging from "strongly disagree" (the least) to "strongly agree" (the most), a great majority of the questions used a 5-point rating system. Measures of content validity and internal consistency reliability were used to determine the questionnaire's reliability and validity. The judgment method ensured the legitimacy of the content. In accordance with the study's suggestion [46], a group of six professionals with expertise in the relevant field (practitioners in the field of education) rated the questionnaire on criteria including relevance, representativeness, specificity, and clarity using a 5-point scale. Cohen's coefficient kappa (k), which was recommended in research [47], was used to assess the degree of expert agreement. The questionnaire's kappa (k) value was 0.87 (significance 0.05∗∗), indicating a high degree of agreement [48]. Additionally, Cronbach's Alpha coefficient is taken into account [49] to measure the dependability of the items-internal consistency. With a Cronbach's Alpha coefficient of 0.803, the results indicated that the questionnaire's internal consistency reliability was acceptable [50]. All 95 participants in the study had participated in a physical survey to gather the data, which was then entered into Excel for further analysis. This study used purposive sampling with a focus on university teachers in Bangladesh. Purposive sampling, or judgment sampling, is a nonrandom technique in qualitative and quantitative research where participants are deliberately selected based on specific qualities, expertise, and characteristics relevant to the research focus [51]. This systematic approach to participant selection ensured that the insights acquired were relevant to the experiences and perspectives of this particular group by concentrating on individuals who had a direct bearing on the study's setting. The research focused on Bangladeshi university teachers to gather detailed and context-specific data that would enhance the relevance and breadth of the result of this study in the local academic community.

### 2.6 Data analysis

Two steps of data analysis were performed. Initially, the descriptive analysis was done using Pandas and NumPy libraries of Python in COLAB. Subsequently, the key "driving" constructs influencing teachers' job satisfaction were analyzed utilizing PLS-SEM [38], an advanced second-generation multivariate analysis technique, in SmartPLS (version: 4) [52]. The approach consists of two models: the measurement model (referred to as the outer model) and the structural model (also referred to as the inner model). The primary use of PLS-SEM is to examine intricate correlations between observable and latent variables. Researchers typically employ this strategy to create ideas in exploratory research and investigate the intricate correlations among several variables at the same time [38]. For this study, PLS-SEM was chosen because it provides researchers with a wider scope to explore and try out various configurations [53], particularly when the sample size is smaller [38]. In addition, according to the study [54], PLS-SEM is more suited than other techniques (such as CB-SEM) when the objective is to predict the important "driver" constructs. In this study, the major parameter settings of SmartPLS are as follows: (i) Algorithm to handle missing data: None; Use Lochmueller settings? No; Initial weights: 1; Max. number of iterations: 300; Data metric: Mean 0, Var 1; Stop Criterion (10^-X): 7; and Weighting scheme: Path.

### 2.7 Ethical considerations

The necessary approval was obtained from the Research Ethics Committee (REC) of the Faculty of Science and Information Technology (FSIT) of Daffodil International University (Reference: REC-FSIT-2022-07-15). The purpose of the study and research objectives were informed to the participants before data collection. At the same time, privacy, ethical factors, and confidentiality of the participants were adequately addressed to avoid confidentiality dilemmas. The REC of FSIT monitored the whole survey to make sure the data collection team followed the approved ethical procedures.

## 3 Results

### 3.1 Participant’s demographic profile

The demographic profile of the sample (N = 95) consists of five components: Gender, Age, Educational Qualification, Designation, and Teaching Experience. Table 1 describes the demographic analysis of the Participant sample.

### 3.2 Evaluation of measurement model

#### 3.2.1 Convergent validity

Convergent validity refers to the degree to which a measure is correlated with other measures of the same concept in accounting for the variability in its measurements. It suggests that measures with comparable or identical concepts should exhibit significant associations [44]. To ensure convergent validity, scholars often use indicators' outer loadings and average variance extracted (AVE) [38,55]. Prior research has suggested that the outer loadings and AVE values should not be less than 0.708 and 0.50, respectively [[38], [43], [55]].

According to Table 2, the AVEs of all constructs were greater than 0.5, indicating that each construct accounts for over 50 % of the variance in its constituent parts. On the other hand, CGO4 (outer loading = −0.029), LED2 (outer loading = 0.616), REC1 (outer loading = 0.237), and SFB3 (outer loading = 0.680) did not meet the suggested threshold. However, removing all four of the indicators resulted in an increase in their respective AVEs, as shown in Table 3.

**Table 2:** Participant’s demographic profile.

Profile	Frequency (N = 95)	Percentage (%)
**Gender**		
Male	60	63 %
Female	35	37 %
**Age**		
21–30	62	65 %
31–40	21	22 %
41–50	7	7 %
51–60	4	4 %
>60	1	1 %
**Educational Qualification**		
Bachelor's Degree	33	35 %
Master's Degree	42	44 %
PhD Enrolled	10	11 %
PhD	10	11 %
**Designation**		
Lecturer	65	68 %
Senior Lecturer	7	7 %
Assistant Professor	12	13 %
Associate Professor	6	6 %
Professor	5	5 %
**Teaching Experience**		
≤5	64	67 %
6–15	25	26 %
16–30	4	4 %
>30	2	2 %

**Table 3:** Validity and reliability of the measurement model keeping all indicators.

Constructs	Indicators	Convergent Validity					Internal Consistency Reliability		
		Std. Dev.	Mean	Loading	AVE	P value	Cronbach’s Alpha	Composite reliability (rho_a)	Composite reliability (rho_c)
Career Growth and Opportunity	CGO1	0.054	0.820	0.830	0.527	0.000	0.634	0.792	0.764
	CGO2	0.023	0.897	0.899		0.000			
	CGO3	0.061	0.777	0.780		0.000			
	CGO4	0.190	−0.024	−0.029		0.439			
Leadership	LED1	0.035	0.847	0.849	0.646	0.000	0.861	0.896	0.900
	LED2	0.117	0.606	0.616		0.000			
	LED3	0.069	0.804	0.809		0.000			
	LED4	0.055	0.832	0.835		0.000			
	LED5	0.023	0.883	0.880		0.000			
Relationships with Colleagues	RC1	0.083	0.766	0.779	0.630	0.000	0.714	0.716	0.836
	RC2	0.058	0.853	0.861		0.000			
	RC3	0.103	0.739	0.736		0.000			
Recognition	REC1	0.288	0.264	0.237	0.614	0.205	0.634	0.939	0.796
	REC2	0.040	0.910	0.929		0.000			
	REC3	0.018	0.950	0.960		0.000			
Salary and Financial Benefits	SFB1	0.040	0.870	0.876	0.693	0.000	0.848	0.849	0.899
	SFB2	0.041	0.861	0.865		0.000			
	SFB3	0.080	0.681	0.680		0.000			
	SFB4	0.024	0.889	0.891		0.000			
Teacher's Job Satisfaction	TJS1	0.034	0.878	0.881	0.801	0.000	0.876	0.876	0.924
	TJS2	0.022	0.908	0.909		0.000			
	TJS3	0.022	0.895	0.896		0.000			
Working Environment	WE1	0.013	0.940	0.941	0.885	0.000	0.870	0.870	0.939
	WE2	0.014	0.940	0.941		0.000			

After excluding the specified indicators, the AVE value of their corresponding constructs increased. As shown in Table 3, all of the AVE values exceed the threshold, it can be concluded that the measurement model has achieved convergent validity.

#### 3.2.2 Discriminant validity

Discriminant validity pertains to how distinct a construct is from other constructs in empirical terms [38,55]. Its main goal is to make sure that an indicator and reflecting component are strongly associated. Scholars typically use three methods to evaluate discriminant validity: (i) examining cross-loadings of indicators, (ii) applying the Fornell-Larcker criterion [56], and (iii) the Heterotrait-Monotrait Ratio of Correlations (HTMT) [57]. Based on [38], an indicator's loadings on its associated construct should exceed the loadings of all other indicators on other constructs; otherwise, discriminant validity may have an issue. The cross-loadings of each indicator that met the specified threshold are shown in Table 4.

**Table 4:** Validity and reliability of the measurement model after removing the indicators with low outer loadings.

Constructs	Indicators	Convergent Validity					Internal Consistency Reliability		
		Standard deviation (STDEV)	Mean	Loading	AVE	P values	Cronbach's alpha	Composite reliability (rho_a)	Composite reliability (rho_c)
Career Growth and Opportunity	CGO1	0.054	0.825	0.831	0.706	0	0.792	0.814	0.878
	CGO2	0.022	0.897	0.895		0			
	CGO3	0.054	0.79	0.791		0			
Leadership	LED1	0.034	0.858	0.859	0.726	0	0.875	0.897	0.914
	LED3	0.067	0.815	0.82		0			
	LED4	0.062	0.819	0.823		0			
	LED5	0.017	0.906	0.905		0			
Relationships with Colleagues	RC1	0.083	0.766	0.779	0.63	0	0.714	0.716	0.836
	RC2	0.058	0.853	0.861		0			
	RC3	0.103	0.739	0.736		0			
Recognition	REC2	0.032	0.929	0.933	0.899	0	0.889	0.939	0.947
	REC3	0.009	0.964	0.962		0			
Salary and Financial Benefits	SFB1	0.022	0.924	0.927	0.837	0	0.903	0.909	0.939
	SFB2	0.024	0.919	0.922		0			
	SFB4	0.022	0.895	0.895		0			
Teacher's Job Satisfaction	TJS1	0.035	0.877	0.88	0.801	0	0.876	0.876	0.924
	TJS2	0.022	0.909	0.91		0			
	TJS3	0.022	0.896	0.896		0			
Working Environment	WE1	0.013	0.94	0.941	0.885	0	0.87	0.87	0.939
	WE2	0.014	0.94	0.94		0			

A more careful method of evaluating discriminant validity is the Fornell-Larcker criterion [56]. The primary comparison made by this technique is between the correlations between constructs and the square root of AVE values [38]. The square root of AVE for each construct must be bigger than the construct with which it has the strongest correlation in the same model in order to demonstrate discriminant validity [[38], [56], [58]]. Table 5 presents the corresponding results, consistent with the recommended approach.

**Table 5:** Cross-loadings of indicators of the measurement model.

	Career Growth and Opportunity	Leadership	Relationships with colleagues	Recognition	Salary and Financial Benefits	Teacher's Job Satisfaction	Working Environment
CGO1	**0.831**	0.537	0.331	0.318	0.381	0.441	0.372
CGO2	**0.895**	0.637	0.567	0.482	0.457	0.616	0.553
CGO3	**0.791**	0.661	0.395	0.59	0.411	0.514	0.435
LED1	0.635	**0.859**	0.588	0.593	0.374	0.543	0.562
LED3	0.536	**0.82**	0.564	0.566	0.409	0.477	0.517
LED4	0.646	**0.823**	0.491	0.555	0.36	0.535	0.607
LED5	0.667	**0.905**	0.567	0.577	0.39	0.703	0.518
RC1	0.188	0.345	**0.779**	0.414	0.275	0.333	0.524
RC2	0.298	0.362	**0.861**	0.38	0.311	0.446	0.563
RC3	0.653	0.732	**0.736**	0.479	0.275	0.542	0.382
REC2	0.499	0.623	0.478	**0.933**	0.367	0.422	0.523
REC3	0.554	0.645	0.546	**0.962**	0.424	0.556	0.544
SFB1	0.448	0.367	0.318	0.377	**0.927**	0.365	0.453
SFB2	0.383	0.341	0.275	0.349	**0.922**	0.404	0.403
SFB4	0.527	0.504	0.397	0.421	**0.895**	0.445	0.437
TJS1	0.562	0.568	0.513	0.446	0.449	**0.88**	0.54
TJS2	0.549	0.625	0.533	0.454	0.291	**0.91**	0.495
TJS3	0.587	0.615	0.51	0.506	0.454	**0.896**	0.528
WE1	0.588	0.653	0.618	0.561	0.446	0.549	**0.941**
WE2	0.446	0.554	0.523	0.499	0.44	0.546	**0.94**

Last but not least, the HTMT value estimates the discriminant validity between two constructs. The SmartPLS software documentation also recommends using the HTMT test for discriminant validation [59]. When the HTMT score is less than 0.90, discriminant validity between the two reflective notions has been attained. The results in Table 6 demonstrate that each construct has the HTMT value that is suggested.

**Table 6:** The Fornell-Larcker criterion of constructs of the measurement model.

	Career Growth and Opportunity	Leadership	Recognition	Relationships with colleagues	Salary and Financial Benefits	Teacher's Job Satisfaction	Working Environment
Career Growth and Opportunity	**0.84**						
Leadership	0.732	**0.852**					
Recognition	0.558	0.669	**0.948**				
Relationships with colleagues	0.527	0.646	0.544	**0.794**			
Salary and Financial Benefits	0.499	0.447	0.42	0.364	**0.915**		
Teacher's Job Satisfaction	0.633	0.674	0.524	0.579	0.446	**0.895**	
Working Environment	0.55	0.642	0.563	0.607	0.471	0.582	**0.941**

As a result, the measurement model's discriminant validity was validated through the cross-loading method, the Fornell-Larcker criterion, and the Heterotrait-Monotrait Ratio of Correlations (HTMT).

#### 3.2.3 Internal consistency reliability

The degree of resemblance between the observed indicator variables in a test or survey is referred to as internal consistency. Cronbach's alpha and composite reliability are the common measures used to evaluate internal consistency reliability.

A Cronbach's alpha value of at least 0.7 is generally considered indicative of good internal consistency, whereas a value below 0.7 is considered questionable. As shown in Table 7, all constructs have Cronbach's alpha values of at least 0.7, indicating a good internal consistency of the measurement model.

**Table 7:** Heterotrait-monotrait ratio of correlations (HTMT) value of constructs of the measurement model.

	Career Growth and Opportunity	Leadership	Recognition	Relationships with colleagues	Salary and Financial Benefits	Teacher's Job Satisfaction	Working Environment
Career Growth and Opportunity							
Leadership	0.871						
Recognition	0.653	0.761					
Relationships with colleagues	0.613	0.764	0.662				
Salary and Financial Benefits	0.581	0.5	0.463	0.445			
Teacher's Job Satisfaction	0.748	0.756	0.583	0.698	0.496		
Working Environment	0.65	0.741	0.639	0.778	0.531	0.667	

Composite reliability is a more appropriate metric than Cronbach's alpha because it ranks indicators based on their individual reliability [38]. Composite reliability scores range from 0 to 1, with higher scores indicating greater reliability. Therefore, composite reliability >0.60 is considered acceptable in exploratory research [38,43]. All of the constructions, as shown in Table 7, were over the suggested threshold level (>0.60). This ensures that the sample has the necessary internal consistency and dependability.

### 3.3 Evaluation of structural model

The second stage of analyzing PLS-SEM findings involves evaluating the structural model [58]. To assess the structural model, five criteria recommended by Refs. [[38], [45], [55]] should be evaluated, which include collinearity assessment, structural model path coefficients, effect size (f2), coefficient of determination (R2), and predictive relevance (Q2).

#### 3.3.1 Collinearity assessment

To ensure that the OLS regressions do not affect the structural model's path coefficients, it is necessary to investigate any collinearity issues [45]. The variance inflation factor (VIF) value of a construct is used to assess collinearity. As suggested by Refs. [45,60], a construct's tolerance (VIF) value should be between 0.20 and 5 to avoid collinearity issues. When the VIF value is between 1 and 5, the constructs are considered moderately correlated [[61], [62]]. Table 8 displays the measured VIF values, and all of them fall within the suggested range of 1–5, indicating no collinearity issues. The five aspects to be evaluated to analyze the structural model, according to Refs. [38,55], and [45], are collinearity assessment, path coefficients of the structural model, coefficient of determination (R2), effect size (f2), and predictive relevance (Q2).

**Table 8:** Internal consistency reliability of the measurement model.

	Cronbach's alpha	Composite reliability (rho_a)	Composite reliability (rho_c)
Career Growth and Opportunity	0.792	0.814	0.878
Leadership	0.875	0.897	0.914
Recognition	0.889	0.939	0.947
Relationships with colleagues	0.714	0.716	0.836
Salary and Financial Benefits	0.903	0.909	0.939
Teacher's Job Satisfaction	0.876	0.876	0.924
Working Environment	0.870	0.870	0.939

#### 3.3.2 Structural model path coefficients

In this study, the use of a one-tailed test for evaluating path coefficients in PLS-SEM is justified by the directional nature of our hypotheses. One-tailed tests are appropriate when prior research and theoretical frameworks provide a strong basis for expecting a specific direction of the relationship between variables. For instance, previous studies have consistently indicated positive effects of leadership and career growth opportunities on job satisfaction among teachers, justifying the expectation of similar directional impacts in our study [63,64]. Additionally, PLS-SEM's default setting for one-tailed tests aligns with its typical application in exploratory and predictive modeling contexts, where directional hypotheses are often tested [65]. This approach enhances the statistical power to detect effects in the expected direction, providing a more precise assessment of the hypothesized relationships.

**Fig. 3:**
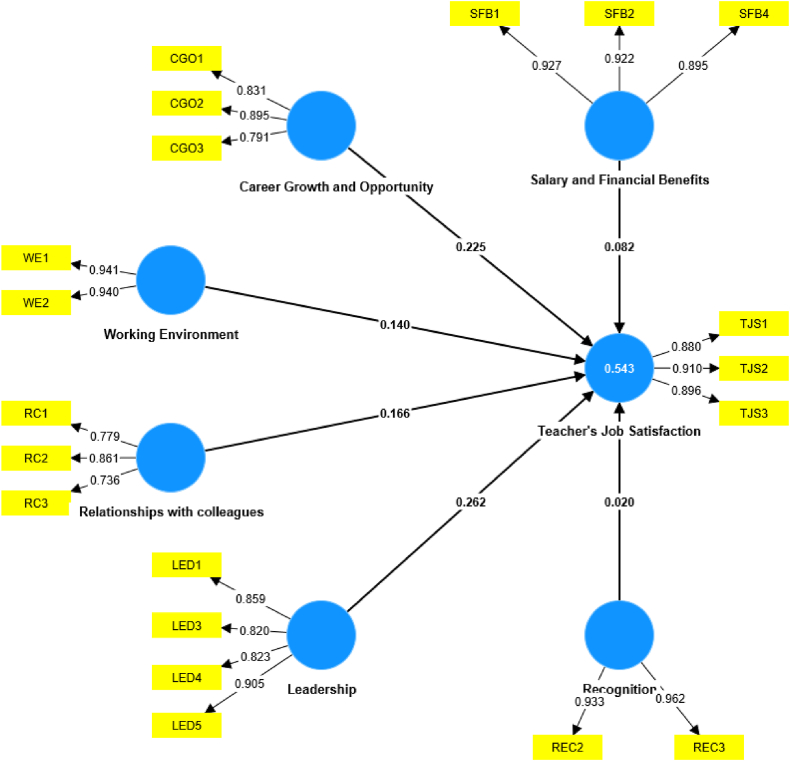
Final model.

Table 9 delineates the effects and statistical significance of factors influencing teachers' job satisfaction, organized in accordance with their respective rankings. Table 9 and Fig. 3 demonstrate that all the constructs positively influence teachers' job satisfaction. Among them, three constructs are found to be statistically significant. Leadership (β = 0.262, t = 1.757, p < 0.10), Career Growth and Opportunity (β = 0.225, t = 2.023, p < 0.10), and Relationships with colleagues (β = 0.166, t = 1.519, p < 0.10) have a positive and significant effect on teachers' job satisfaction, which supports H5, H2, and H6, respectively.

**Table 9:** Collinearity assessment.

Constructs	VIF	Higher than 0.10 and lower than 5
Career Growth and Opportunity	2.371	Yes
Leadership	3.235	Yes
Recognition	1.99	Yes
Relationships with colleagues	1.961	Yes
Salary and Financial Benefits	1.454	Yes
Working Environment	2.082	Yes

#### 3.3.3 Coefficient of determination (R2)

The coefficient of determination (R2) is frequently used by scholars to measure a model's explanatory power. The range of the R2 value is 0–1, with higher values suggesting better explanatory power. R2 values of 0.75, 0.50, and 0.25 for endogenous constructs are regarded as significant, reasonable, and poor, respectively, by Ref. [38]. In accordance with Table 5, the R2 value for teacher job satisfaction is 0.543, which indicates that the construct has a moderate R2 and the models have a strong explanatory power as suggested by Ref. [45].

#### 3.3.4 Effect size (f2)

To evaluate the relative influence of exogenous constructs on endogenous constructs, researchers often use effect size, also known as f2. The f2 values range from 0 to 1, with higher values indicating greater impact. According to Refs. [38,45], an f2 value of 0.02, 0.15, and 0.35 indicates a small, medium, and large effect, respectively, of an exogenous construct on an endogenous construct. Table 10 indicates that Career Growth and Opportunity, Leadership, Relationships with Colleagues, and Working Environment have a small effect on Teacher's Job Satisfaction, while Salary and Financial Benefits and Recognition have no effect (see Table 11).

**Table 10:** Path coefficient.

Path Coefficient	Coefficient (Original)	Coefficient (Mean)	Std. Dev.	T Statistics	P Values	Rank
Leadership - > Teacher's Job Satisfaction	0.262	0.289	0.149	1.757	**0.039**	**1**
Career Growth and Opportunity - > Teacher's Job Satisfaction	0.225	0.217	0.111	2.023	**0.022**	**2**
Relationships with colleagues - > Teacher’s Job Satisfaction	0.166	0.156	0.109	1.519	**0.064**	**3**
Working Environment - > Teacher's Job Satisfaction	0.14	0.141	0.132	1.057	0.145	**4**
Salary and Financial Benefits - > Teacher's Job Satisfaction	0.082	0.072	0.099	0.823	0.205	**5**
Recognition - > Teacher's Job Satisfaction	0.02	0.018	0.096	0.208	0.418	**6**
	**R-square**	**R-square adjusted**				
Teacher's Job Satisfaction	0.543	0.512

**Table 11:** Effect size.

Constructs	f2	effect size
Career Growth and Opportunity	0.047	small
Leadership	0.047	small
Recognition	0	no effect
Relationships with colleagues	0.031	small
Salary and Financial Benefits	0.01	no effect
Teacher's Job Satisfaction	–	–
Working Environment	0.02	small

#### 3.3.5 Predictive relevance (Q2)

In order to determine the cross-validated redundancy measures for each endogenous construct, the blindfolding procedure was used to calculate the predictive relevance (Q2) value. The blindfolding technique is a sample reuse method that allows for the calculation of Stone-Geisser's Q2 value, which is a criterion for evaluating the cross-validated predictive relevance of the PLS path model. However, the most recent version of SmartPLS (version 4) no longer supports the blindfolding method or algorithm because it does not provide an out-of-sample assessment of predictive power.

#### 3.3.6 PLSpredict (PLS path model estimations)

Table 12 presents the PLSpredict path model estimates, based on the algorithm proposed by Shmueli et al. [52,29], which assesses predictive performance using Q^2^predict, RMSE (Root Mean Square Error), and MAE (Mean Absolute Error) metrics. The construct evaluated include TJS. The Q^2^predict value, indicating predictive accuracy, was 0.100, demonstrating robust predictive performance. The RMSE value, which capture the variability in prediction errors, show that the model delivers the good accuracy with the lower RMSE of 0.711. The MAE values, reflecting the average size of prediction errors, highlight the TJS construct as having the small error at 0.783. Overall, the model is characterized by its highest Q^2^predict and lowest RMSE, indicating that this construct offers superior predictive performance according to the PLSpredict algorithm.

**Table 12:** PLSpredict LV summary.

	Q^2^ predict	RMSE	MAE
Teacher’s Job Satisfation (TJS)	0.100	0.711	0.783

## 4 Discussion

The study examined the effects of Leadership, Career Growth and Opportunity, Relationships with Colleagues, Working Environment, Salary and Financial Benefits and Recognition constructs on job satisfaction among university teachers in Bangladesh. The study proposes a new model to evaluate teacher job satisfaction at the university level, using partial least squares structural equation modeling (PLS-SEM). The validity and reliability of the reflective measurement model were established through Table 2, Table 3, Table 4, Table 5, Table 6, Table 7, while Table 8, Table 9, Table 10 use collinearity analysis, path coefficients, model explanatory power, effect size, and predictive relevance to evaluate the structural model. The study's findings are presented in Fig. 3.

From the structural model evaluation, it is apparent that Leadership (β = 0.262, t = 1.757, p < 0.10) exerts a positive and significant impact on Teacher’s Job Satisfaction, marking a substantive finding with broader implications. The positive coefficient indicates that effective leadership within educational institutions contributes significantly to the enhancement of teacher job satisfaction. This resonates with the outcomes of studies conducted by Refs. [13,14], wherein the authors underscored the pivotal role of leadership in fostering a positive work environment for teachers. Their conclusions align with our findings, emphasizing that a positive relationship between institutional administration and teachers is integral to minimizing teacher dissatisfaction. Moreover, the rank of Interpersonal relationships with administrators as 11th among the motivational factors in the study by Mertler et al. [17] further substantiates the impact of leadership on the motivational dynamics of teaching. This highlights the complex link between the degree of job satisfaction among teachers and the nature of their interpersonal connections with administrators. Similarly, findings from studies conducted in the context of Bangladesh [18,19] reinforce the universal relevance of leadership as a determinant of teacher job satisfaction. These collective insights emphasize that leadership styles tailored to the local cultural and institutional context can significantly influence job satisfaction, which may explain the consistency of this finding across different settings. In essence, the positive and significant impact of Leadership identified in our study underscores the critical role of effective leadership practices in nurturing a conducive and satisfying professional environment for teachers. These collective insights substantiate the theoretical and practical significance of leadership as a pivotal factor influencing teacher job satisfaction, thereby enriching our understanding within the broader spectrum of educational research.

Additionally, this study reveals that Career Growth and Opportunity has a positive and significant impact (β = 0.225, t = 2.023, p < 0.10) on teachers’ job satisfaction. This finding is not merely a statistical observation but holds substantive implications for the educational landscape. The positive coefficient suggests that as opportunities for career advancement increase, teachers' job satisfaction also experiences a concurrent elevation. This aligns with the insights from BURÓN et al. [12], emphasizing the critical role of Promotion and Growth Opportunities in shaping teachers' satisfaction. Moreover, the correlation identified in our study resonates with Saiti et al. [13], emphasizing the intrinsic link between job satisfaction and promotional opportunities. Ghenghesh [14] further enriches this understanding by highlighting the pivotal role of training and development opportunities, asserting that they constitute a cornerstone in fostering teachers’ professional satisfaction. Additionally, our findings parallel those of Mertler et al. [17], indicating that Greater opportunities for advancement rank prominently among the factors influencing teachers to remain in the teaching profession. The alignment with these studies suggests that career development opportunities are universally important across different contexts, although the specific nature of these opportunities may vary. These collective insights underscore the universal significance of Career Growth and Opportunity as a central tenet shaping teachers’ job satisfaction, emphasizing its practical and theoretical relevance within the broader educational framework.

Furthermore, this study reveals that Relationships with Colleagues (β = 0.166, t = 1.519, p < 0.10) wield a positive and significant impact on Teacher’s Job Satisfaction, imparting meaningful insights into the dynamics of teacher well-being. The positive coefficient suggests that fostering positive and collaborative relationships with colleagues contributes significantly to the overall job satisfaction of teachers. This corroborates the findings of Mertler et al. [17], where Interpersonal relationships with colleagues ranked prominently as the 4th motivational factor in teaching jobs. Our study extends this understanding by delving into the broader implications of such relationships, indicating that Greater opportunities for collaboration with colleagues were also ranked 4th among the factors influencing teachers to remain in the teaching profession. Moreover, the positive correlation between teachers’ job satisfaction and job performance, as identified by Amin et al. [11], further underscores the profound impact of Relationships with Colleagues on teachers’ overall professional experience. The significance of collegial relationships highlights the importance of a supportive and collaborative work environment, which can enhance job satisfaction and professional performance. The universality of these findings is underscored by the resonance of similar patterns in studies conducted in Bangladesh [[18], [19], [21]], where Relationships with Colleagues emerged as a consistently impactful factor in measuring teacher job satisfaction. This suggests that interpersonal dynamics within academic institutions are crucial across different cultural contexts, reflecting the intrinsic human need for supportive professional relationships. In sum, our study contributes to the growing body of knowledge by affirming the significance of positive interpersonal dynamics among colleagues as a fundamental determinant of teachers’ job satisfaction, thereby enriching the broader discourse on teacher well-being and satisfaction within the educational milieu.

In addition, our findings indicate that Working Environment (β = 0.14, t = 1.057, p > 0.10) exerts a positive impact on university Teacher’s job satisfaction, although the impact did not reach statistical significance. This observation gains resonance when considered alongside recent research [15], emphasizing the multifaceted nature of factors contributing to Bangladeshi teachers' job satisfaction. The positive influence of the Working Environment, identified in our study, aligns with similar research [[18], [19], [21]] that underscores the pivotal role of the working environment in shaping teachers’ job satisfaction. Moreover, the study by Mertler et al. [17] provides nuanced insights by ranking Working Conditions as the 8th motivational factor in teaching jobs. The acknowledgment that a Lack of a supportive work environment is ranked 4th among the reasons to leave the teaching profession further underscores the intricate relationship between the quality of the working environment and its impact on teachers' professional satisfaction and retention. The non-significant impact in our study could be attributed to the varying conditions across different universities, suggesting a need for more standardized improvements in the working environment. While the impact of the Working Environment in our study did not achieve statistical significance, the collective evidence from existing research and our findings emphasizes the enduring importance of cultivating a positive and supportive workplace atmosphere for university teachers. This highlights the need for administrators to consider not only tangible factors like salary and promotion opportunities but also intangible elements such as the working environment to foster a conducive and satisfying professional atmosphere for educators.

Conversely, this study indicates that Salary and Financial Benefits (β = 0.082, t = 0.823, p > 0.10) exhibit a positive impact on teachers’ job satisfaction, albeit not reaching statistical significance. This nuanced finding stands in contrast to recent studies [[11], [12], [13], [14], [15], [16], [17]] that unequivocally highlight the crucial role of Salary and Financial Benefits in shaping teachers’ job satisfaction. The non-significant impact in our study prompts a critical reflection on the preferences and priorities of university teachers in Bangladesh. One plausible interpretation could be that university teachers in Bangladesh place a higher emphasis on intrinsic motivational factors such as Career Growth, Leadership, and Interpersonal Relationships with colleagues, surpassing the relative significance of financial considerations. The incongruity with existing literature might signify a nuanced shift in the drivers of job satisfaction among university educators in Bangladesh, suggesting that non-monetary aspects weigh more heavily in their professional contentment. Contrary findings in similar studies conducted in Bangladesh [[18], [19], [20], [21]], where salary emerged as a pivotal factor influencing job satisfaction, add an additional layer to the discussion. This dichotomy could imply a temporal evolution in the priorities of university teachers in Bangladesh, reflecting a gradual inclination towards non-financial aspects such as career growth and interpersonal relationships, indicative of a broader shift in the professional landscape over time. This divergence from established findings could be influenced by cultural and socio-economic factors unique to Bangladesh, where intrinsic rewards might be perceived as more fulfilling in the academic profession. Additionally, the evolving economic conditions and educational policies in Bangladesh could be shifting the emphasis away from financial incentives towards personal and professional development. In sum, our study contributes to the ongoing discourse on the determinants of teachers’ job satisfaction by shedding light on a potentially evolving landscape, wherein non-monetary factors may be gaining prominence in the professional considerations of university teachers in Bangladesh.

Finally, our findings suggest that Recognition (β = 0.02, t = 0.208, p > 0.10) imparts a positive impact on Teacher’s job satisfaction, despite not reaching statistical significance. This observation gains significance when contextualized within the broader framework of research on teachers’ job satisfaction. The congruence with the study by Ghenghesh [14], where Recognition ranked as the 3rd source of teachers’ job satisfaction, underscores the persistent acknowledgment of recognition as a pivotal factor in shaping educators' contentment. Similarly, the alignment of our findings with the study by Mertler et al. [17], positioning Recognition as the 3rd motivational factor in teaching jobs, further corroborates the enduring importance of acknowledgment and appreciation in the professional lives of teachers. This consistency across studies emphasizes the recurrent theme that, while not statistically significant in our study, Recognition remains an integral aspect of the complex tapestry of teacher job satisfaction. The non-significant result may reflect specific cultural or institutional practices in Bangladesh that affect how recognition is perceived and valued among university teachers. Iwu et al.'s [16] conclusion regarding the importance of Recognition, among other factors, in determining teachers’ job satisfaction adds depth to our understanding. The non-significant positive impact identified in our study aligns with the collective wisdom of existing research, emphasizing the significance of Recognition alongside other factors. This suggests that, despite not being statistically significant in this study, recognition is likely to be an important element of job satisfaction that warrants further investigation within different cultural and organizational contexts. In sum, our study contributes to the ongoing discourse on teacher job satisfaction by reaffirming the persistent positive impact of Recognition, albeit not statistically significant in isolation. Recognition remains a valuable aspect within the multifaceted dynamics of teachers’ professional fulfillment, suggesting its enduring importance in the complex interplay of factors influencing teacher well-being.

## 5 Recommendations and policy implications

The results of this study suggest the following recommendations and policy implications for improving the job satisfaction of university teachers in countries like Bangladesh.

• **Career Growth and Opportunities:** The University must support faculty members in attending professional development opportunities and provide guidance and mentorship to newly appointed teachers while establishing transparent criteria for promotion and tenure and offering support to achieve these goals. To effectively implement these opportunities, universities can create structured career development plans tailored to individual faculty members, establish partnerships with other institutions for exchange programs, and regularly review and update promotion criteria to reflect current academic standards. These strategies not only enhance job satisfaction but also foster long-term commitment and reduce turnover. Supported by empirical data, specialized career development programs designed to meet the specific requirements of faculty members greatly enhance both work satisfaction and retention rates [66]. In line with critical thoughts on internationalization and cross-institutional cooperation in higher education, faculty exchange programs promote research collaboration and professional growth [67].
• **Leadership:** The University administration should promote a positive and collaborative relationship with teachers, encouraging open communication and a culture of mutual respect and professionalism to create a supportive and productive working environment. Universities can offer leadership training programs for administrators that focus on inclusive and transformational leadership styles, and set up regular town hall meetings to facilitate direct dialogue between faculty and leadership. Implementing these practices can lead to a more engaged and motivated faculty, improving overall institutional performance. Supported by empirical evidence, leadership training programs that are grounded in the ideas of transformational leadership have been shown to enhance faculty involvement and institutional performance [68]. Established communication channels like as town hall meetings facilitate open and collaborative interaction between professors and administration [69].
• **Relationships with Colleagues:** The University must encourage open communication and collaboration among teachers to foster interdisciplinary research while promoting mutual respect and addressing any conflicts in a prompt and fair manner to maintain a positive and harmonious working environment. Creating faculty lounges or collaborative spaces, organizing regular interdisciplinary seminars and workshops, and establishing a formal peer mediation program for conflict resolution can significantly improve collegial relationships. Enhanced collaboration and reduced conflicts contribute to a more cohesive and productive academic community. Bolstered by empirical evidence, collaborative spaces and multidisciplinary seminars have shown the ability to enhance faculty relations and research output [70]. Furthermore, peer mediation programs facilitate dispute resolution and foster a constructive work atmosphere [71].
• **Salary and Financial Benefits:** The University must evaluate the performance of teachers and offer merit-based salary increases accordingly, while also conducting regular market surveys to ensure competitive salaries among comparable institutions. Implementing a transparent performance appraisal system and providing regular feedback can ensure fair evaluations. Additionally, establishing a salary review committee that includes faculty representatives can help align compensation with market standards. These measures can enhance job satisfaction and retention by ensuring faculty members feel valued and fairly compensated. The implementation of transparent performance assessment methods and consistent feedback greatly improves teacher satisfaction [72]. The use of pay review committees has been shown to synchronize remuneration with prevailing market norms and enhance employee retention [73].
• **Working Environment:** The University must ensure fair and respectful treatment of all teachers and provide a safe and healthy working environment, offering flexible work arrangements and support to manage workloads. Additionally, the University should avoid imposing arbitrary rules that do not serve a meaningful purpose. Introducing well-defined policies for work-life balance, such as flexible scheduling and remote work options, and setting up support services like counseling and wellness programs can create a more supportive environment. Regularly reviewing and streamlining administrative processes can also reduce unnecessary stress. Such initiatives can lead to a healthier, more productive faculty. Supported by empirical data, work-life balance initiatives, such as the implementation of flexible work arrangements, have been associated with a decrease in faculty burnout and an enhancement in job satisfaction [74]. Academic institutions that provide counseling and wellness services have been shown to have increased rates of faculty retention [75].
• **Recognition:** The University should arrange programs to acknowledge and reward faculty members for their accomplishments and publicize these achievements to enhance their reputation. Additionally, the University should offer support to teachers seeking external recognition for their achievements. Developing an annual awards ceremony, highlighting achievements in internal newsletters, and providing resources for grant applications and award nominations can effectively recognize faculty contributions. This recognition can boost morale and incentivize high performance, contributing to greater job satisfaction and professional fulfillment. Research has shown that recognition initiatives, including prizes and internal acknowledgment, may significantly increase teacher morale and improve work performance and productivity [76]. Facilitating professors in their pursuit of funding and external recognition has the potential to enhance professional happiness and research productivity [77].

## 6 Conclusion

Higher education is undoubtedly one of the most important key factors for the development of any nation. Since Bangladesh is a developing country, the higher education sector is being enriched day by day. Thousands of students are graduating every year in Bangladesh. To ensure the best quality higher education, it is very important to ensure proper job satisfaction of the teachers. This study's findings underscore the distinctive factors influencing job satisfaction among university teachers in Bangladesh, offering critical insights for the development of effective policies and administrative practices. The results contribute significantly to the broader discourse on educational quality and teacher retention, particularly in the context of developing nations. Our research bridges the gap in quantitative analysis, providing a comprehensive understanding that can inform future strategies for enhancing teacher job satisfaction globally. Utilizing PLS-SEM to explore the important "driver" constructs of job satisfaction in university teachers, this study found career growth and opportunities, leadership, and relationship with colleagues have a significant positive influence, and salary and financial benefits, recognition, and working environment have a positive but not significant influence on teacher’s job satisfaction in university level. Surprisingly, salary and financial benefits do not have a significant impact on job satisfaction, which signifies that university teachers are more influenced by factors like career growth rather than financial benefits. On the other hand, career growth and opportunities seem to be the most important factor for university teachers’ job satisfaction in the context of Bangladesh. Finally, this study authenticates the definite link between CGO, LED, REC, RC, SFB, WE, and TJS (see Figure). The overall findings should aid the universities to implement synchronized strategies to promote teachers’ job satisfaction.

## 7 Study limitations and research directions

The study's limitations must be taken into account when interpreting the results, despite providing significant insights into employee retention strategies. Firstly, the cross-sectional nature of the study limits the ability to establish causality between the studied factors and job satisfaction. While longitudinal studies would provide a more comprehensive understanding of the dynamics of job satisfaction over time, the cross-sectional design was chosen due to time constraints and lack of financial support. Secondly, there is a potential for response bias given the method of data collection (physical surveys). Participants might have provided socially desirable answers or been reluctant to express dissatisfaction, which could influence the results. Thirdly, this study only examined six factors affecting job satisfaction in university teachers and did not explore all possible predictors. Future studies should consider including a broader range of factors to provide a more comprehensive analysis. Fourthly, due to the lack of previous research in Bangladesh, there was insufficient comparison with findings from similar studies in this context. Finally, the universities were chosen randomly, so demographic effects were not observed. To improve representation, future studies should include a larger number of universities from various locations across the country. Additionally, the cultural and contextual specificity of the study, conducted in the unique context of Bangladesh, may limit the applicability of the findings to other settings with different cultural, economic, and educational systems.

## CRediT authorship contribution statement

**Md Abdul Hye Zebon:** Writing – review & editing, Writing – original draft, Visualization, Validation, Methodology, Formal analysis, Conceptualization. **Abdus Sattar:** Supervision, Project administration. **Md Sazzadur Ahamed:** Investigation, Data curation.

## Data availability

The data that support the findings of this study are available on request from the corresponding author. The data are not publicly available due to privacy or ethical restrictions.

## Declaration of competing interest

The authors declare that they have no known competing financial interests or personal relationships that could have appeared to influence the work reported in this paper.

## References

[1] SDG Resources for Educators – Quality Education, UNESCO. [Online]. Available: https://en.unesco.org/themes/education/sdgs/material/04 [Accessed: 14-May-2022].

[2] Education Statistics Datatopics.worldbank.org. https://datatopics.worldbank.org/education/wRsc/classification.

[3] Goczek Ł., Witkowska E., Witkowski B. (Jun. 2021). How does education quality affect economic growth?. Sustainability.

[4] Grant C. (Mar. 03, 2017). https://opendocs.ids.ac.uk/opendocs/handle/20.500.12413/13117.

[5] Colclough C. (Mar. 1982). The impact of primary schooling on economic development: a review of the evidence. World Dev..

[6] Arias Rafael, Giménez Gregorio, Sánchez Leonardo (2016). Impact of education on poverty reduction in Costa Rica: a regional and urban-rural analysis. Contemporary Rural Social Work Journal.

[7] Mayer D.P., Mullens J.E., Moore M.T., Ralph J. (2000).

[8] Ortan F., Simut C., Simut R. (Dec. 2021). Self-efficacy, job satisfaction and teacher well-being in the K-12 educational system. Int. J. Environ. Res. Publ. Health.

[9] Holmqvist M. (May 2019). Lack of qualified teachers: a global challenge for future knowledge development. Teacher Education in the 21st Century.

[10] Molero Jurado M. del M., Pérez-Fuentes M. del C., Atria L., Oropesa Ruiz N.F., Gázquez Linares J.J. (Apr. 2019). Burnout, perceived efficacy, and job satisfaction: perception of the educational context in high school teachers. BioMed Res. Int..

[11] Amin M. (Aug. 2015). Relationship between job satisfaction, working conditions, motivation of teachers to teach and job performance of teachers in MTs, serang, banten. J. Manag. Sustain..

[12] Gamero Burón C., Lassibille G. (Jun. 2016). Job satisfaction among primary school personnel in Madagascar. J. Dev. Stud..

[13] Saiti A., Papadopoulos Y. (Jan. 2015). School teachers' job satisfaction and personal characteristics. Int. J. Educ. Manag..

[14] Ghenghesh P. (Jan. 2013). Job satisfaction and motivation - what makes teachers tick?. British Journal of Education, Society & Behavioural Science.

[15] Sultana A., Sarker M.N.I., Prodhan A.S. (2017). Job satisfaction of public and private primary school teachers of bogra district in Bangladesh. Journal of Sociology and Anthropology.

[16] Iwu C., Ezeuduji I., Iwu I., Ikebuaku K., Tengeh R. (Feb. 2018). Achieving quality education by understanding teacher job satisfaction determinants. Soc. Sci..

[17] Mertler C.A. (Mar. 2016). Should I stay or should I go? Understanding teacher motivation, job satisfaction, and perceptions of retention among Arizona teachers. International Research in Higher Education.

[18] Ali T., Akhter I. (2009). Job satisfaction of faculty members in private universities -in context of Bangladesh. Int. Bus. Res..

[19] Asadujjaman Md, Or Rashid Md H., Nayon Md A.A., Biswas T.K., Arani M., Billal M.M. (Dec. 2020). 2020 Sixth International Conference on E-Learning (Econf).

[20] Tabassum A. (2012). Interrelations between quality of work life dimensions and faculty member job satisfaction in the Private Universities of Bangladesh. Eur. J. Bus. Manag..

[21] Masum A.K.M., Azad Md A.K., Beh L.-S. (Feb. 2015). Determinants of academics' job satisfaction: empirical evidence from private universities in Bangladesh. PLoS One.

[22] Toropova A., Myrberg E., Johansson S. (Jan. 2020). Teacher job satisfaction: the importance of school working conditions and teacher characteristics. Educ. Rev..

[23] Crisci A., Sepe E., Malafronte P. (Mar. 2018). What influences teachers' job satisfaction and how to improve, develop and reorganize the school activities associated with them. Qual. Quantity.

[24] Liu Y., Bellibaş M.Ş., Gümüş S. (Mar. 2020). The effect of instructional leadership and distributed leadership on teacher self-efficacy and job satisfaction: mediating roles of supportive school culture and teacher collaboration. Educ. Manag. Adm. Leader.

[25] Jnr B.A. (2021). An exploratory study on academic staff perception towards blended learning in higher education. Educ. Inf. Technol..

[26] Madigan D.J., Kim L.E. (2021). Towards an understanding of teacher attrition: a meta-analysis of burnout, job satisfaction, and teachers' intentions to quit. Teach. Teach. Educ..

[27] Maleka M.J., Paul-Dachapalli L., Ragadu S.C., Schultz C.M., Van Hoek L. (2020). Performance management, vigour, and training and development as predictors of job satisfaction in low-income workers. SA J. Hum. Resour. Manag..

[28] Jnr B.A. (2021). Institutional factors for faculty members' implementation of blended learning in higher education. Educ + Train.

[29] Shmueli G., Ray S., Estrada J.M.V., Chatla S.B. (Oct. 2016). The elephant in the room: predictive performance of PLS models. J. Bus. Res..

[30] List of Private Universities, University Grants Commission of Bangladesh. [Online]. Available: http://www.ugc-universities.gov.bd/private-universities.

[31] List of Public Universities, University Grants Commission of Bangladesh. [Online]. Available: http://www.ugc-universities.gov.bd/public-universities.

[32] Hossain M.Y., Hossain M.N. (2016). Job satisfaction of private university teachers in Bangladesh. European Business and Management.

[33] Setia M. (2016). Methodology series module 3: cross-sectional studies. Indian J. Dermatol..

[34] Levin K.A. (2006). Study design III: cross-sectional studies. Evid. Base Dent..

[35] Wang X., Cheng Z. (2020). Cross-sectional studies. CHEST Journal.

[36] “47th Annual Report 2020,” University Grants Commission of Bangladesh. [Online]. Available: https://ugc.portal.gov.bd/. [Accessed: 1-April-2023].

[37] do Valle P.O., Assaker G. (Feb. 2015). Using partial least squares structural equation modeling in tourism research. J. Trav. Res..

[38] Hult G.T.M., Ringle C.M., Sarstedt M., Hair J.F. (2016).

[39] Ferine K.F., Aditia R., Rahmadana M.F., Indri (Jul. 2021). An empirical study of leadership, organizational culture, conflict, and work ethic in determining work performance in Indonesia's education authority. Heliyon.

[40] Kock N., Hadaya P. (Nov. 2016). Minimum sample size estimation in PLS-SEM: the inverse square root and gamma-exponential methods. Inf. Syst. J..

[41] Kock N. (Dec. 2018). Minimum sample size estimation in PLS-SEM: an application in tourism and hospitality research. Applying Partial Least Squares in Tourism and Hospitality Research.

[42] Faul F., Erdfelder E., Buchner A., Lang A.-G. (Nov. 2009). Statistical power analyses using G∗Power 3.1: tests for correlation and regression analyses. Behav. Res. Methods.

[43] Ashraf M.A., Ahmed H. (Feb. 2022).

[44] Sukendro S. (Nov. 2020). Using an extended Technology Acceptance Model to understand students' use of e-learning during Covid-19: Indonesian sport science education context. Heliyon.

[45] Hair J., Hult G.T.M., Ringle C.M., Sarstedt M. (2021).

[46] Haynes S.N., Richard D.C.S., Kubany E.S. (Sep. 1995). Content validity in psychological assessment: a functional approach to concepts and methods. Psychol. Assess..

[47] Boateng G.O., Neilands T.B., Frongillo E.A., Melgar-Quiñonez H.R., Young S.L. (2018). Best practices for developing and validating scales for health, social, and behavioral research: a primer. Front. Public Health.

[48] McHugh M.L. (2012). Interrater reliability: the kappa statistic. Biochem. Med..

[49] Kawakami N. (May 2020). Internal consistency reliability, construct validity, and item response characteristics of the Kessler 6 scale among hospital nurses in Vietnam. PLoS One.

[50] Tavakol M., Dennick R. (Jun. 2011). Making sense of Cronbach's alpha. Int. J. Med. Educ..

[51] Etikan I. (2016). Comparison of convenience sampling and purposive sampling. Am. J. Theor. Appl. Stat..

[52] Ringle C., Wende S., Becker J. (2015). SmartPLS 3.3.9, SmartPLS. https://www.smartpls.com/.

[53] Dash G., Paul J. (Dec. 2021). CB-SEM vs PLS-SEM methods for research in social sciences and technology forecasting. Technol. Forecast. Soc. Change.

[54] Choosing between pls-SEM and CB-sem – SmartPLS, Smartpls.com. https://www.smartpls.com/documentation/choosing-pls-sem/choosingbetween-pls-sem-and-cb-sem/.

[55] Hair J.F., Risher J.J., Sarstedt M., Ringle C.M. (Jan. 2019). When to use and how to report the results of PLS-SEM. Eur. Bus. Rev..

[56] Fornell C., Larcker D.F. (Feb. 1981). Evaluating structural equation models with unobservable variables and measurement error. J. Market. Res..

[57] Discriminant validity assessment and heterotrait-monotrait ratio of correlations (HTMT). https://www.smartpls.com/documentation/algorithms-and-techniques/discriminant-validity-assessment.

[58] Sarawa D.I., Mas’ud A. (Jan. 2020). Strategic public procurement regulatory compliance model with mediating effect of ethical behavior. Heliyon.

[59] Discriminant validity assessment and heterotrait-monotrait ratio of correlations (HTMT), SmartPLS, Smartpls.com. https://www.smartpls.com/documentation/algorithms-and-techniques/discriminant-validity-assessment.

[60] Becker J.-M., Ringle C.M., Sarstedt M., Völckner F. (May 2014). How collinearity affects mixture regression results. Market. Lett..

[61] Variance inflation factor, Statistics how to. https://www.statisticshowto.com/variance-inflation-factor.

[62] Ahmmed S., Saha J., Tamal M.A. (Oct. 2022). An empirical study for determining the quality indicators for the primary and secondary school of Bangladesh: a structural equation modeling approach. Heliyon.

[63] Cohen J. (2013).

[64] Henseler J., Ringle C.M., Sarstedt M. (2014). A new criterion for assessing discriminant validity in variance-based structural equation modeling. J. Acad. Market. Sci..

[65] Han J., Yin H., Wang J., Zhang J. (2019). Job demands and resources as antecedents of university teachers' exhaustion, engagement and job satisfaction. Educ. Psychol..

[66] Bland C.J., Seaquist E., Pacala J.T., Center B., Finstad D. (May 2002). One Schoolʼs strategy to assess and improve the vitality of its faculty. Acad. Med..

[67] Robson S., Wihlborg M. (Mar. 2019). Internationalisation of higher education: impacts, challenges and future possibilities. Eur. Educ. Res. J..

[68] Avolio B.J., Gardner W.L. (2005). Authentic leadership development: getting to the root of positive forms of leadership. Leader. Q..

[69] Spreitzer G., Porath C. (2012). Creating sustainable performance. Harv. Bus. Rev..

[70] Amey M.J., Brown D.F. (2005). Interdisciplinary collaboration and academic work: a case study of a university-community partnership. N. Dir. Teach. Learn..

[71] Johnson D.W., Johnson R.T. (Dec. 1996). Conflict resolution and peer mediation programs in elementary and secondary schools: a review of the research. Rev. Educ. Res..

[72] Dasanayaka C.H., Abeykoon C., Ranaweera R.A.A.S., Koswatte I. (Oct. 2021). The impact of the performance appraisal process on job satisfaction of the academic staff in higher educational institutions. Educ. Sci..

[73] Kramer B., Bosman J. (May 2024).

[74] Daniel S., Sonnentag S. (Mar. 2015). Crossing the borders: the relationship between boundary management, work–family enrichment and job satisfaction. Int. J. Hum. Resour. Manag..

[75] Greenhaus J.H., Beutell N.J. (Jan. 1985). Sources of conflict between work and family roles. Acad. Manag. Rev..

[76] Deci E.L., Koestner R., Ryan R.M. (2001). Extrinsic rewards and intrinsic motivation in education: reconsidered once again. Rev. Educ. Res..

[77] Motivating Factors and Obstacles behind Grant Research: The Case of a Teaching-Focused State College,” www.srainternational.org. https://www.srainternational.org/blogs/srai-jra1/2021/03/23/motivating-factors-and-obstacles-behind-grant-rese.

