# Supplementary material for: An empirical study of exploring the predictors of university teachers’ job satisfaction in Bangladesh: A structural equation modeling approach
Source: Heliyon. 2025 Jan 6;11(2):e41740. doi: 10.1016/j.heliyon.2025.e41740 (PMC11770486; doi:10.1016/j.heliyon.2025.e41740)
Supplement: Multimedia component 1 [file mmc1.docx]

**University Teachers' Job Satisfaction**

Teachers are requested to carefully go through the questionnaire and answer appropriately. Your feedback in this regard will be very useful and highly appreciated. The data collected through this questionnaire will not be shared with a third party and will be considered as confidential. The identity of the participant will not be disclosed.

**Section 1**

**Demographic Information**

1. Gender (লিঙ্গ)
   - Male
   - Female
2. Age (বয়স)
   - Input your age in years (e.g. 35)
3. Designation (পদবি)
   - Professor
   - Associate Professor
   - Assistant Professor
   - Senior Lecturer
   - Lecturer
4. Educational Qualification (শিক্ষাগত যোগ্যতা)
   - Please enter your last educational qualification (e.g. B.Sc./M.Sc./PhD Candidate/PhD etc.)
5. Department (বিভাগ)
   - Please enter the name of your department
6. Teaching Experience (শিক্ষকতার অভিজ্ঞতা)
   - Please enter your teaching experience in years (e.g. 2 years, 1 year, 0 year)

**Section 2**

**Salary and Financial Benefits**

1. I am fairly paid for my job (আমি আমার কাজের জন্য যথেষ্ট পারিশ্রমিক পাই)
   - Strongly Disagree
   - Disagree
   - Neutral
   - Agree
   - Strongly Agree
2. I am satisfied with my current salary (আমি আমার বর্তমান বেতন নিয়ে সন্তুষ্ট)
   - Strongly Disagree
   - Disagree
   - Neutral
   - Agree
   - Strongly Agree
3. I am satisfied with the bonus and other financial benefits in this university (আমি এই বিশ্ববিদ্যালয়ে বোনাস এবং অন্যান্য আর্থিক সুবিধা নিয়ে সন্তুষ্ট)
   - Strongly Disagree
   - Disagree
   - Neutral
   - Agree
   - Strongly Agree
4. I am satisfied with the overall pay structure (আমি সামগ্রিক বেতন কাঠামো নিয়ে সন্তুষ্ট)
   - Strongly Disagree
   - Disagree
   - Neutral
   - Agree
   - Strongly Agree

**Section 3**

**Career Growth and Opportunity**

1. I have the opportunity to learn new things and develop new skills (আমার কাছে এখানে নতুন জিনিস শেখার এবং নতুন দক্ষতা বিকাশের সুযোগ রয়েছে)
   1. Strongly Disagree
   2. Disagree
   3. Neutral
   4. Agree
   5. Strongly Agree
2. I am satisfied with the opportunities I receive (আমি যে সুযোগগুলো পেয়েছি তাতে আমি সন্তুষ্ট)
   1. Strongly Disagree
   2. Disagree
   3. Neutral
   4. Agree
   5. Strongly Agree
3. I am satisfied with my chances of promotions in this University (আমি এই বিশ্ববিদ্যালয়ে আমার পদোন্নতির সম্ভাবনা নিয়ে সন্তুষ্ট)
   1. Strongly Disagree
   2. Disagree
   3. Neutral
   4. Agree
   5. Strongly Agree
4. How many research papers do you publish on a yearly basis? (আপনি বছরে কতগুলো গবেষণাপত্র প্রকাশ করেন?)

**Section 4**

**Working Environment**

1. I am satisfied with my working environment (আমি আমার কাজের পরিবেশ নিয়ে সন্তুষ্ট)
   1. Strongly Disagree
   2. Disagree
   3. Neutral
   4. Agree
   5. Strongly Agree
2. My working environment is safe and comfortable (আমার কাজের পরিবেশ নিরাপদ এবং আরামদায়ক)
   1. Strongly Disagree
   2. Disagree
   3. Neutral
   4. Agree
   5. Strongly Agree

**Section 5**

**Relationships with colleagues**

1. I have good relationship with my colleague (আমার সহকর্মীর সাথে আমার ভালো সম্পর্ক আছে)
   1. Strongly Disagree
   2. Disagree
   3. Neutral
   4. Agree
   5. Strongly Agree
2. I like the people I work with (আমি যাদের সাথে কাজ করি তাদের পছন্দ করি)
   1. Strongly Disagree
   2. Disagree
   3. Neutral
   4. Agree
   5. Strongly Agree
3. I have a good understanding with the leadership of this University (এই বিশ্ববিদ্যালয়ের নেতৃত্বের সঙ্গে আমার ভালো বোঝাপড়া আছে)
   1. Strongly Disagree
   2. Disagree
   3. Neutral
   4. Agree
   5. Strongly Agree

**Section 6**

**Leadership**

1. The University administration encourages and supports me (বিশ্ববিদ্যালয় প্রশাসন আমাকে উৎসাহিত করে এবং সমর্থন করে)
   1. Strongly Disagree
   2. Disagree
   3. Neutral
   4. Agree
   5. Strongly Agree
2. I am not bound by too many rules (আমি খুব বেশি নিয়মে আবদ্ধ নই)
   1. Strongly Disagree
   2. Disagree
   3. Neutral
   4. Agree
   5. Strongly Agree
3. My issues are resolved whenever I report the authorities (আমি যখনই কর্তৃপক্ষকে রিপোর্ট করি তখনই আমার সমস্যাগুলি সমাধান করা হয়)
   1. Strongly Disagree
   2. Disagree
   3. Neutral
   4. Agree
   5. Strongly Agree
4. I am given enough freedom to decide how I do my work (আমি কীভাবে আমার কাজ করব তা সিদ্ধান্ত নেওয়ার জন্য আমাকে যথেষ্ট স্বাধীনতা দেওয়া হয়েছে)
   1. Strongly Disagree
   2. Disagree
   3. Neutral
   4. Agree
   5. Strongly Agree
5. I have full confidence in the leadership of this University (এই বিশ্ববিদ্যালয়ের নেতৃত্বের প্রতি আমার পূর্ণ আস্থা রয়েছে)
   1. Strongly Disagree
   2. Disagree
   3. Neutral
   4. Agree
   5. Strongly Agree

**Section 7**

**Recognition**

1. How many times does your organization arrange a recognition event on a yearly basis? (আপনার প্রতিষ্ঠান বার্ষিক ভিত্তিতে কতবার একটি স্বীকৃতি ইভেন্টের ব্যবস্থা করে?) (e.g. 1, 2, 3 etc.)
2. I get proper recognition that I deserve (আমি উপযুক্ত স্বীকৃতি পাই যা আমি প্রাপ্য)
   1. Strongly Disagree
   2. Disagree
   3. Neutral
   4. Agree
   5. Strongly Agree
3. I am satisfied with the recognition and supports that I receive (আমি যে স্বীকৃতি এবং সমর্থন পেয়েছি তাতে আমি সন্তুষ্ট)
   1. Strongly Disagree
   2. Disagree
   3. Neutral
   4. Agree
   5. Strongly Agree

**Section 8**

**Overall Satisfaction**

1. Being a teacher of this University was a right decision for me (এই বিশ্ববিদ্যালয়ের শিক্ষক হওয়া আমার জন্য একটি সঠিক সিদ্ধান্ত ছিল)
   1. Strongly Disagree
   2. Disagree
   3. Neutral
   4. Agree
   5. Strongly Agree
2. I have a long-range vision for my career in this University (এই বিশ্ববিদ্যালয়ে আমার কর্মজীবনের জন্য আমার দীর্ঘ পরিসরের দৃষ্টিভঙ্গি রয়েছে)
   1. Strongly Disagree
   2. Disagree
   3. Neutral
   4. Agree
   5. Strongly Agree
3. Considering everything, are you satisfied with your job? (সবকিছু বিবেচনা করে, আপনি কি আপনার কাজ নিয়ে সন্তুষ্ট?)
   1. Strongly Disagree
   2. Disagree
   3. Neutral
   4. Agree
   5. Strongly Agree
